# Renal safety profile of STB in virologically suppressed subjects from two randomized phase 3b switch trials

**DOI:** 10.7448/IAS.17.4.19807

**Published:** 2014-11-02

**Authors:** Iain Reeves, Martin Fisher, Stephen Kegg, Jose Arribas, Lauren Dau, Will Garner, Ivan Walker, Thai Nguyen

**Affiliations:** 1 Homerton Sexual Health Services, Homerton University Hospital, London, UK; 2 Brighton and Sussex University Hospital NHS Trust, Department of HIV, Brighton, UK; 3 The Trafalgar Clinic, Queen Elizabeth Hospital, London, UK; 4 Consulta de Medicina Interna II, Hospital Universitario La Paz, Madrid, Spain; 5 HIV Medical Affairs, Gilead Sciences, Foster City, USA; 6 Department of Biostatistics, Gilead Sciences, Foster City, USA

## Abstract

**Introduction:**

Cobicistat, a component of stribild (STB), is known to inhibit renal creatinine secretion. A detailed analysis of the renal safety profile of STB in two Phase 3b switch studies of virologically-suppressed individuals on stable therapy: STRATEGY(S)-PI (STB vs a RTV-boosted protease inhibitor [PI] with emtricitabine and tenofovir DF [FTC/TDF]); and STRATEGY(S)-NNRTI (STB versus a non-nucleoside reverse transcriptase inhibitor [NNRTI] with FTC/TDF) is herein described.

**Materials and Methods:**

Baseline eGFR ≥70 mL/min was an inclusion criterion. The renal safety profile of STB was examined by baseline eGFR through week 48 (i.e., changes in eGFR, renal tubular laboratory abnormalities, investigator-reported renal adverse events leading to discontinuation and unreported subclinical proximal renal tubulopathy [PRT]). Subclinical PRT was defined as a confirmed serum-creatinine increase ≥0.4 mg/dL and two or three markers of renal tubular dysfunction (hypophosphatemia, normoglycemic glycosuria, proteinuria) occurring at the same visit at least once and with no alternative etiologies.

**Results:**

In S-PI, 433 subjects (STB 293; PI 140) and in S-NNRTI, 434 subjects (STB 291; NNRTI 143) were randomized and treated. Most (>85%) STB subjects had a baseline eGFR ≥90 mL/min. STB subjects with baseline eGFR <90 mL/min had smaller declines in eGFR compared to those with baseline eGFR ≥90 mL/min and similar occurrences of renal tubular laboratory abnormalities (Table 1). Rate of renal adverse events leading to study drug discontinuation were similar for the STB group (one PRT in a subject with baseline tubular laboratory abnormalities consistent with underlying PRT and one isolated increase in serum creatinine) and PI group (one isolated decrease in eGFR); none in the NNRTI group. The case of PRT improved after study drug discontinuation. There were no cases of unreported subclinical PRT in any group.

**Conclusions:**

In this virologically suppressed patient population, the renal safety of STB did not differ by baseline eGFR. The renal discontinuation rate was low in the STB group, similar to the RTV-boosted PI group, and consistent with published historical rates.

**Table 1:** Changes in eGFR at week 48 and tubular laboratory abnormalities by baseline eGFR

S-PI Study Arm	STB	STB	PI+RTV	PI+RTV
Baseline eGFR (mL/min) category	<90 (n=40)	≥90 (n=253)	<90 (n=27)	≥90 (n=113)
Changes from baseline at week 48: eGFR (mL/min), median (IQR)	−3.8 (−8.1 to 1.2)	−8.9 (−16.9 to 0.4)	−0.2 (−4.5 to 5.0)	0.5 (−9.1 to 7.3)
Hypophosphatemia*	0	0.4% (1)	0	0
Normoglycemic glycosuria*	0	0	0	0
Proteinuria*	5.0% (2)	9.5% (24)	11.1% (3)	7.1% (8)
**S-NNRTI Study Arm**	**STB**	**STB**	**PI+RTV**	**PI+RTV**
Baseline eGFR (mL/min) category	<90 (n=43)	≥90 (n=248)	<90 (n=20)	≥90 (n=123)
Changes from baseline at week 48: eGFR (mL/min), median (IQR)	−8.0 (−11.6 to 0.9)	−12.6 (−21.2 to −6.3)	7.4 (1.9 to 14.4)	−1.7 (−10.9 to 5.8)
Hypophosphatemia*	4.7% (2)	0	0	1.6% (2)
Normoglycemic glycosuria*	2.3% (1)	1.2% (3)	0	0
Proteinuria*	9.3% (4)	6.9% (17)	10% (2)	4.9% (6)
				

* ≥1-grade increase from baseline observed at two consecutive post-baseline visits (within 30 days after last dose date).

